# Report of Diseases in the Public and Private Practice of One of the Physicians of the Finsbury Dispensary, from the 20th of September to the 20th of October

**Published:** 1805-11-01

**Authors:** J. Reid

**Affiliations:** Grenville Street, Bruuswick Square


### *Report of Diseases in the public and private Practice of one of the Physicians of the Finsbury Dispensary, from the 20th of September to the 20th of October.*

| | | | |
|---|---|---|---|
| Rheumatismus - - - | 11 | Amenorrhœa - - - - | 7 |
| Catarrhus - - - - - | 15 | Menorrhagia - - - - | 4 |
| Phthisis pulmonalis - - | 9 | Tussis - - - - - - - | 12 |
| Ophthalmia - - - - | 2 | Dyspepsia - - - - - | 8 |
| Ephemera - - - - - | 2 | Colica - - - - - - - | 1 |
| Scarlatina - - - - - - | 3 | Hydrops Pectoris - - | 3 |
| Morbi infantiles - - - | 16 | Pneumatisis - - - - | 2 |
| Morbi cutanei - - - | 12 | Asthenia - - - - - - | 18 |

Early in the last month, the Reporter was roused from his repose at a nocturnal hour by a call to a patient laboring under a violent attack of internal pain, which was accompanied by every other circumstance that could concur to menace the approaching danger of inflammation in the stomach, or some part of the intestinal canal.

This was a case of peculiar delicacy and danger. The extreme degree of general debility, strikingly indicated by a depression of the spirits and pulse, as well as other circumstances, when occurring simultaneously with local irritation, or a partial excess of excitement, often involve the the Practitioner in embarrassment with regard to the measures which ought to be immediately adopted. The evacuation of blood, which the inflammation seems to require, is calculated to exaggerate that debility which is a still more important and alarming symptom.

In such instances of exigence and peril, purgatives, especially in the form of enema, whilst they in a great degree answer the purpose of venæsection, are not attended by those risks and inconveniences that are apt to follow the latter process. By a powerful and efficacious application of this kind, the patient, in the instance alluded to, was relieved not long after the moment of its administration.

A military officer, who had been repeatedly in the West Indies, and two campaigns in Holland during the last war, applied lately to the Reporter. He was strongly affected with a disorder of the nervous system, not indeed amounting to, but in some degree partaking of, the nature of mental derangement. He had been what is called a high liver, and in other respects licentiously luxurious. He has since acquired more accurate and better regulated habits. But his debilitated constitution still continues to suffer from the results of juvenile dissipation. Some tonics of a medicinal nature were prescribed, connected with the habitual use of the Shower-bath, which, with a proper attention to physical and moral regimen, appeared not unlikely, in the course of time, to invigorate and restore, in a certain degree, the decayed energies of his frame.

A case has recently occurred of a person afflicted with dyspepsia, particularly marked with a *bad breath.* This last symptom he lamented as having essentially interfered with his most important prospects and purposes in life. As is usual where the stomach is ill qualified to discharge its duty, there appeared an hypochondriacal irritability and depression of the nervous system, which not improbably induced the patient both to exaggerate his disease, as well as the unfortunate and unpleasant consequences arising from it. The Reporter convinced the patient that his ailment was not in his mouth, but in the stomach, and that by correcting the depraved condition of that important organ by certain regulations of diet and pharmaceutical preparations, he might be relieved in time from that offensive exhalation, the actual or fancied existence of which he so feelingly deplored. In connection with this case, it is worthy of remark, how much the state of the breath is affected by that of the spirits.

How

How long will it be before even the appointed and professional guardians of the physical constitution shall be brought duly to appreciate the almost immensurable influence which the mental part of our frame, in an advanced and ameliorated state of society, unintermittingly exercises over its health, its preservation, and decay.

The savage, the rustic, the mechanical drudge, or the infant whose faculties have not had time to unfold themselves, or which, in physiological language, have not as yet been *secreted*, may for the most part be regarded as machines regulated principally by physical agents. But man, matured, civilized, and by due culture lifted to his destined level in the scale of being, partakes more of a moral than of an animal character, and is in consequence to be worked upon by remedies that apply themselves to his imagination, his passions, or his judgment, still more than by those that are directed immediately to the parts and functions of his material organization.

Nearly every month the writer of this article has been irresistibly led to touch upon this subject, because every month he has met with fresh and frequent instances confirming the truth, and upon his own mind impressing more deeply the importance of his sentiments with regard to it.

J. REID.

*Grenville Street, Brunswick Square, Oct. 27,* 1805.

